# Feasibility and Acceptability of a Healthy Nordic Diet Intervention for the Treatment of Depression: A Randomized Controlled Pilot Trial

**DOI:** 10.3390/nu13030902

**Published:** 2021-03-10

**Authors:** Julia A. Sabet, Moa S. Ekman, A. Sofia Lundvall, Ulf Risérus, Ulrica Johansson, Åsa Öström, Viola Adamsson, Yang Cao, Mussie Msghina, Robert J. Brummer

**Affiliations:** 1 Faculty of Medicine and Health, School of Medical Sciences, Örebro University, Södra Grev Rosengatan 32, 70362 Örebro, Sweden; moae28@hotmail.com (M.S.E.); sofia.lundvall@hotmail.se (A.S.L.); mussie.msghina@oru.se (M.M.); robert.brummer@oru.se (R.J.B.); 2 School of Hospitality, Culinary Arts and Meal Science, Örebro University, 71202 Grythyttan, Sweden; asa.ostrom@oru.se; 3 Department of Public Health and Caring Sciences, Clinical Nutrition and Metabolism, Uppsala University, 75122 Uppsala, Sweden; ulf.riserus@pubcare.uu.se (U.R.); viola.adamsson@hufb.se (V.A.); 4 Department of Clinical Sciences, Paediatrics, Umeå University, 90185 Umeå, Sweden; ulrica.johansson@umu.se; 5 Clinical Epidemiology and Biostatistics, School of Medical Sciences, Örebro University, 70182 Örebro, Sweden; yang.cao@oru.se; 6 Unit of Integrative Epidemiology, Institute of Environmental Medicine, Karolinska Institutet, 17177 Stockholm, Sweden; 7 Department of Clinical Neuroscience, Karolinska Institutet, 17176 Stockholm, Sweden

**Keywords:** depression, major depressive disorder, diet, nutrition, randomized controlled trial, randomized controlled pilot trial, healthy Nordic diet, mental health, palatability, food liking

## Abstract

Healthy diet interventions have been shown to improve depressive symptoms, but there is a need for randomized controlled trials (RCTs) that are double blind and investigate biological mechanisms. The primary objectives of this randomized controlled pilot trial were to test the palatability of the meals and the acceptability of the intervention in preparation for an 8-week RCT in the future, which will investigate whether a healthy Nordic diet improves depressive symptoms in individuals with major depressive disorder, and associated biological mechanisms. Depressed (*n* = 10) and non-depressed (*n* = 6) women and men were randomized to receive either a healthy Nordic diet (ND) or a control diet (CD) for 8 days. Participants were blinded to their diet allocation and the study hypotheses. Health questionnaires were completed before and after the intervention and, throughout the study, questionnaires assessed participants’ liking for the meals, their sensory properties, adherence, and open-ended feedback. In the ND group, 75% of participants consumed only the provided foods, as instructed, compared to 50% of CD participants. The meals of both diets, on average, received good ratings for liking and sensory properties, though the ND ratings were somewhat higher. Overall, results were positive and informative, indicating that the planned RCT will be feasible and well-accepted, with some proposed modifications.

## 1. Introduction

Depressive disorders are common and are a leading cause of disability worldwide [1]. Major depressive disorder (MDD) is characterized by a persistent depressed mood, loss of interest or pleasure, changes in appetite and sleep, loss of energy, feelings of worthlessness, concentration problems, and suicidal thoughts. A substantial proportion of MDD patients fail to respond to conventional treatments or to achieve remission, which is the ultimate goal of treatment [2,3,4]. Therefore, there is a need for additional effective treatments for depression, given either alone or in combination with currently available treatments, and to identify the biological mechanisms and patient-specific factors that predict treatment response.

Depression is associated with poor diet quality, i.e., a diet that is high in processed foods, refined grains and added sugars, and low in fruits, vegetables and whole grains [5,6,7]. A healthy diet, i.e., one that is abundant in fruits, vegetables, and whole grains, and low in red meat, processed foods, and added sugars, has been consistently and robustly shown to be associated with a reduced risk of depression [8,9,10]. Evidence is emerging that, in addition to its preventive effect, a healthy diet may also be an effective treatment strategy for depression. Two randomized controlled trials (RCTs) investigating a Mediterranean-style diet as a treatment for depression have shown that 12 weeks of dietary support in the form of counseling, recipes, and food hampers resulted in a substantially greater improvement in depressive symptoms and increased remission compared to a social support control group [11,12]. A third RCT, employing a similar 3-week diet intervention also showed a significantly greater improvement in depressive symptoms compared to the control group, who were given no instructions regarding diet and asked to return after 3 weeks for follow up [13]. Although the exact biological mechanisms relating diet to depression are unknown, the evidence, mainly from preclinical studies, suggests that the pathways of inflammation, oxidative stress, epigenetics, the gut microbiota, the hypothalamic-pituitary-adrenal (HPA) axis, and metabolic syndrome, among others, play a role [14].

Although these initial RCT results are promising, there is a need for well-controlled trials in which participants are blinded to group allocation and study hypotheses, as this may reduce expectation bias, which is known to be large in trials of MDD [15,16,17]. Furthermore, there is a need to elucidate the biological mechanisms and mode of action. We plan to perform an RCT in which individuals with MDD will be randomized to receive either a healthy Nordic diet or a control diet for 8 weeks; all food will be provided to all participants for the duration of the trial and biological mechanisms will be thoroughly explored. A healthy Nordic diet was chosen because it is beneficial to use native foods familiar to the local population to increase feasibility and tolerability and to reduce the impact on the environment [18,19,20,21,22]. Previous intervention studies involving a healthy Nordic diet have achieved good dietary adherence and resulted in reduced inflammation markers and an improved blood lipid profile, blood pressure, insulin sensitivity, and cognitive function [23,24,25,26,27]. Our proposed RCT will consist of an 8-day rotating meal plan for each group. We performed the present 8-day pilot trial because a study of this nature has never been performed in depressed individuals, nor have there been any studies in any population comparing a healthy Nordic diet with a control diet in which all food is provided to both groups for the duration of the intervention.

The primary objectives of this study were to gain experience in delivering the intervention, as well as to test the acceptability of the intervention and the meals. We aimed to ensure that all meals and the diets as a whole in the future definitive RCT would be palatable and tolerated by participants and that adherence would be good, thereby ensuring the feasibility of the study and the integrity of our findings. As depression is often characterized by reduced appetite, ensuring that the diets have good sensory properties and are well liked would be expected to increase adherence to the diets and the feasibility of the study, since the palatability of foods is reliably associated with an increased appetite and intake [28]. Meals that received negative ratings in the pilot study could be modified or replaced and any other feedback about the intervention could be used to improve upon the design of the planned RCT. Another primary objective was to investigate the extent to which participant blinding was achieved, while a secondary objective was to investigate whether any self-reported changes in health were observed. We included both depressed and non-depressed participants in this pilot study in order to generalize our findings to other populations so our proposed diet intervention could be implemented in the treatment of other health conditions besides depression.

## 2. Materials and Methods

### 2.1. Study Design

The present study was a parallel-group randomized controlled pilot trial in which participants received either a healthy Nordic diet (ND) or a control diet (CD) for 8 days. The food was prepared in the kitchen of Örebro University Hospital (Örebro, Sweden) and picked up by participants outside the kitchen at specified times, with 5 to 10 minutes between each participant, beginning the day before the start of the intervention and every 3 days thereafter, for a total of 3 times. Five self-rated questionnaires related to mental and physical health were completed by participants at home 1–2 days before the start of the intervention and on the last evening of the diet intervention; those that were paper based were delivered when food was picked up for the first time and after the completion of the intervention, respectively. Throughout the intervention, food-related questionnaires were filled out online. The present study was conducted according to the principles of Good Clinical Practice (GCP) and the Helsinki Declaration of 1975, as revised in 2013. Approval was obtained by the Swedish Ethical Review Authority prior to commencing recruitment (no. 2020-03735). The intervention is reported according to the guidelines of the Consolidated Standards of Reporting Trials (CONSORT) statement, the extension for randomized pilot and feasibility trials, and the Template for Intervention Description and Replication (TIDieR). This trial is registered at ClinicalTrials.gov (NCT04731454).

### 2.2. Participants

The inclusion criteria were (1) men and women between 18 and 65 years of age, and (2) if depressed, a score between 13 and 34 on the Montgomery–Åsberg Depression Rating Scale, self-rated (MADRS-S) [29], indicating mild or moderate depression. The exclusion criteria were (1) a score of 8 or higher (out of a maximum of 12 points) on a brief diet survey (the retired version of the Swedish Food Agency’s online Matvanekollen [30], conducted via phone interview), indicating a relatively healthy habitual diet, (2) the presence of food allergies, intolerances or sensitivities, (3) consuming any form of special diet that excludes certain foods, for example, a vegetarian or gluten-free diet, and (4) suicidality, indicated by a score of 4 or higher on the MADRS-S suicidality question.

Participants were recruited from the general population of Örebro County, Sweden, by a social media advertisement (Facebook and Instagram). Individuals who were interested in participating or learning more about the study filled out a form online with their contact information and basic eligibility questions, such as age, dietary restrictions, and depression status. Those who reported that they were depressed or unsure were emailed the MADRS-S depression questionnaire [29] to complete online. In a subsequent phone call, the remaining eligibility criteria were assessed, including habitual diet quality and dietary restrictions, and complete information about the study was provided. Complete written information was then provided via email and all participants gave their signed informed consent prior to being enrolled and participating in the study.

### 2.3. Diet Intervention

Publicly available and newly created recipes were used for planning the study meals, including published recipes from a previous healthy Nordic diet intervention [24]. For outcomes to be attributed to differences in diet quality rather than caloric intake or macronutrient distribution, both diets contained an average of 2200 kcal per day and a distribution of carbohydrate, fat and protein of approximately 45% of energy intake (E%), 35 E%, and 20 E%, respectively. The dietary calculations were performed using Dietist Net Pro software (Kost och Näringsdata AB, Bromma, Sweden), which uses data from the Swedish Food Agency food database [31]. Prior to beginning the present intervention, recipes were first taste tested by 4 volunteers each to ensure that they were palatable, as determined by a questionnaire we designed to evaluate the 6 Culinary Success Factors: “(i) name and presentation fit the expectation; (ii) appetizing smell that fits the food; (iii) good balance of flavour components in relation to the food; (iv) presence of umami, also called the fifth basic taste; (v) in mouthfeel a mix of hard and soft textures; and (vi) high flavour richness” [32]. These factors represent the common denominators of renowned chefs’ most successful dishes and are considered to be drivers of liking and palatability. The meals were consumed within 3 days in order to mimic the conditions of the present intervention. If similar negative feedback was provided on an aspect by at least two taste testers, the recipe was modified accordingly. Nine recipes were replaced with new recipes due to extensive flaws and/or being deemed too time consuming or complex to prepare, and these were, in a similar manner, taste tested, evaluated and modified as needed.

In the present pilot trial, all food was provided to participants of both groups for 8 days. Though breakfasts and snacks were repeated 2–3 times, all lunches and dinners were unique. The proposed future 8-week trial will consist of an 8-day meal plan that will be repeated 7 times. Eight days of unique main course meals is assumed to provide sufficient dietary variation since it is likely more variation than the average person is used to consuming. The present pilot trial consisted, therefore, of an 8-day intervention in order to test one full cycle of meals. Participants were instructed to eat every meal until they were full (ad libitum) and to not eat any additional foods besides what was provided in the study. Lunches and dinners were fully prepared and supplied in individually portioned containers, requiring only heating up, the addition of sauces, and/or stirring. Ingredients for breakfasts and two snacks per day were provided with simple instructions for how participants should prepare them. Some food or drink items that would be used more than once were supplied on the first day for the whole study. In addition, extra bread, cheese, and margarine (according to each diet) were provided, and participants were instructed to make sandwiches with these to eat if they were still hungry after eating their meals; they were supplied with more if needed at their next pick-up. Participants could choose when to eat their meals and snacks, but were instructed to eat breakfast, lunch, and dinner in the correct order. The ND group was not provided any drinks, but the CD group was provided with a juice drink, which they were instructed to drink every day. Besides this, both groups were instructed not to drink anything besides water, milk, coffee, tea, and alcohol as they were accustomed to.

Table 1 shows the compositions of each diet. The ND was designed to be as healthy as possible and therefore met and exceeded the Nordic Nutrition Recommendations [33] and contained approximately 85% Nordic ingredients (by weight). It was high in vegetables, legumes, fruits, fish, and whole grains, the majority of which were grains other than wheat, such as barley, oats, and rye. It contained no refined grains, added sugars, red meat or processed meat. Dairy products were low fat, and oils and margarine were 100% plant-based, the most abundant being rapeseed oil. The CD was designed to approximate the average depressed person’s diet in Sweden. Since studies in several countries have shown that depressed people tend to have a lower quality diet than non-depressed people [5,6,7], which is supported by clinical experience in Sweden, the CD was designed to be of slightly lower quality than the average diet in Sweden. The Swedish national dietary survey, Riksmaten 2011 [34], was used, which reports the median intakes of all food groups and nutrients, by different age and sex groups. For each food group, we took the lowest median (e.g., median for young men) to set an approximate amount for the healthy food groups (e.g., fruits, vegetables, whole grains, and fish) and the highest median to set an approximate amount for the unhealthy food groups (e.g., added sugars, red meat, and sausage). The CD was high in red meat, refined grains (mostly wheat), and added sugars, and low in fruits, vegetables, and whole grains.

To blind participants, the meals were named and labeled only with codes, and staple items such as bread, milk, yogurt, cheese, and margarine were removed from their original packaging and supplied in new, unlabeled packaging. Written instructions for meal consumption and preparation (i.e., mixing, heating, etc.) were provided for all meals, in a neutral and generic way, referring to codes, for example, so as not to describe or reveal the contents of the food. Although the CD contained 11 E% added sugars, the main sources of sugar in the diet were breakfast granola, yogurt, a juice drink, ketchup, and sauces, rather than desserts, in order to “hide” the sugars. Although the quantity of vegetables in the CD was small, all lunches and dinners had at least a small portion of vegetables or herbs to give the appearance of healthiness and to add more color. The meals were prepared by four experienced and educated cooks hired specifically for the study. M.S.E. and A.S.L. (BSc in Meal Ecology) planned the meals, performed the nutrient calculations, and coordinated and supervised the meal preparation, packaging, and labeling during the study, under the supervision of J.A.S. (PhD in Nutrition).

### 2.4. Sample Size

As this was a pilot study, a sample size calculation was not performed, and our sample size determination was based on feasibility. Since the original recipes were written for 4 portions, it was most feasible to scale up by multiples of 4. In order to deliver a high-quality intervention and have the capacity to adequately manage all participants in this first performance of the intervention, 16 was determined to be the maximum number of participants. Although a combination of depressed and non-depressed participants was desired for generalizability, we aimed to have a majority of depressed participants in order to achieve our primary objectives. Therefore, 10 depressed and 6 non-depressed participants were enrolled.

### 2.5. Randomization and Blinding

The project leader (J.A.S.) recruited and enrolled the participants. The randomization sequence was computer generated by an independent person (D.R.) who was not otherwise involved in the study. Participants were randomized in block sizes of two to the ND or CD groups (1:1), stratified by gender and depression status (depressed or not). The randomization sequence was entered into an Excel document divided into sheets for each stratum. This document was given to another independent person not involved in the study (J.R.) in order to randomly allocate the participants from the list of enrolled participants provided by J.A.S. J.R. then gave the allocation list to the kitchen staff so that they could package the participants’ food in cooler bags, labeled with their names. At the time of pickup, the kitchen staff handed each participant’s filled and closed bag to J.A.S. inside the kitchen, who handed the bags to the participants outside the kitchen. The kitchen staff were not blinded and did not interact with participants unless there were important questions regarding their food, which were taken via email or phone. J.A.S. was the primary contact person for the participants, and she was blinded to diet allocation throughout the study. J.A.S. also performed the data analyses and remained blinded until the written participant feedback was summarized. Participants were also blinded since they were not informed of the nature of the diets being studied, how many diets there were, and what the study hypotheses were. They were simply told that they would be randomized to a diet and that there were no unusual ingredients or supplements. The participants were scheduled to pick up their food at different times so there was minimal or no interaction between them, and they were instructed not to discuss their food with each other or with J.A.S.

### 2.6. Questionnaires Regarding the Diets and Study

While eating every meal and snack, i.e., 5 times per day, participants filled out a meal evaluation questionnaire online. The questionnaire took approximately 5 minutes to complete and contained original questions on the sensory properties of the meal, similar to those used during the taste testing and inspired by the Culinary Success Factors [32]. These questions were either Likert type or used Just About Right (JAR) scales, which measure the intensity of specific sensory attributes, e.g., “just about right”, “too low” or “too high” taste intensity [35]. JAR scales are commonly used in consumer testing to identify specific attributes that are associated with liking or acceptance. Immediately after finishing the meal, participants answered the remaining questions, including a Likert-type question on portion size and visual analog scale (VAS) items on how full they felt and how healthy they perceived the meal to be. In order to assess the degree to which the meal was liked, the commonly used and validated 7-point hedonic scale was used, rating from “dislike very much” to “like very much” [36]. Participants also answered whether or not they ate everything and, if not, what and approximately how much was remaining and why. Finally, they were free to provide any additional comments about the meal. Every evening before bedtime, participants completed an online questionnaire in which they were asked to report whether they ate any of the extra bread, margarine, or cheese that was provided and any additional non-study foods or drinks. In the CD group, the participants were asked whether they drank a whole portion of juice drink as they were instructed to do. At the end of the study, participants completed an online questionnaire with a combination of multiple-choice, VAS, and open-ended questions regarding their overall impressions of the study and diet as a whole, suggestions for improvement, how easy it was to adhere to the protocol, how easy it would be to adhere for 8 weeks, overall impressions of healthiness and amount of food in the diet, changes in weight and physical and mental health, and other comments. The main objectives of these three questionnaires were to investigate the acceptability and feasibility of the diet intervention.

### 2.7. Health-Related Questionnaires

Although this pilot study was not designed or powered to detect health-related changes, nor were significant changes expected after only 8 days, we included five of the questionnaires that we plan to incorporate in the future definitive RCT for the following reasons: (1) to investigate whether either diet resulted in negative health effects, for example, whether the high-fiber ND caused substantial gut problems; (2) to investigate potential “placebo” effects which could indicate that blinding was successful and that the intervention was well accepted; (3) to gain experience with and test the use of a web-based data capture platform that was new to and never before used at Örebro University; (4) to explore whether any positive effects on physical or mental health or activity level occurred after an 8-day diet intervention. The Work Productivity and Activity Impairment (WPAI) questionnaire [37] determines health-related work productivity and consists of 6 questions on work productivity, absenteeism, and ability to perform daily activities; it has been shown to have good reproducibility. The Gastrointestinal Symptoms Rating Scale (GSRS) [38] consists of 15 questions, each addressing a different gastrointestinal symptom that is rated on a 7-point scale according to the severity of symptoms. The symptoms are divided into five symptom domains, including reflux, abdominal pain, indigestion, diarrhea, and constipation. The GSRS has been shown to be reliable and to have good internal consistency for most domains, but low test–retest reliability [39]. The Frändin–Grimby Physical Activity Scale [40] consists of one 6-level question describing one’s amount of physical activity with regard to household chores, leisure time, and physical training, and has been shown to have good validity. The Montgomery–Åsberg Depression Rating Scale, self-rated (MADRS-S) [29] contains 9 items, corresponding to the main depressive symptoms, which are rated on a scale of 0 to 6 according to severity. The MADRS-S has been found to have good reliability (Cronbach’s alpha = 0.84), to be sensitive to change, and to be a good complement to the clinician-based MADRS [29]; furthermore, there is a high correlation between internet and paper administration of the test (r = 0.84; *p* < 0.001) [41]. The EuroQol 5-level generic health questionnaire (EQ-5D-5L) [42,43] consists of five items related to wellbeing and function and provides a summary index value for each possible health profile based on people’s strength of preference for different health profiles. In addition, participants rate their subjectively experienced overall health status on a VAS ranging from 0 to 100. The EQ-5D-5L has been shown to have good validity and reliability (Cronbach’s alpha = 0.77) in patients with major depression [44]. The MADRS-S and EQ-5D-5L were completed online, and the other three questionnaires were performed on paper and delivered when food was picked up for the first time and at the end of the study when the cooler bags were returned.

All online questionnaires in this study were performed and managed using the Research Electronic Data Capture (REDCap) platform hosted at Örebro University [45,46]. REDCap is a secure, web-based software platform designed to support data capture for research studies, providing (1) an intuitive interface for validated data capture; (2) audit trails for tracking data manipulation and export procedures; (3) automated export procedures for seamless data downloads to common statistical packages; and (4) procedures for data integration and interoperability with external sources. Through REDCap, the participants were scheduled to receive the questionnaires by email at specific times, and reminder emails were sent automatically if the questionnaires were not filled out by a certain time. Basic information about the participants was also entered into REDCap, including age, sex, weight, depression status, and diet allocation.

### 2.8. Data Analyses

Outcomes were analyzed and reported in terms of the mean and standard deviation (SD) or 95% confidence interval (CI), median with interquartile range (IQR) or 95% CI, or count (percentage). In order to check for differences in baseline characteristics between groups, the mean, SD and a t-test were used for data that met the assumptions; the median, IQR and Mann–Whitney U test were used for ordinal or non-normally distributed data. The meal evaluation questionnaire results were analyzed by first calculating the median of each participant’s responses for each question across all meals and snacks of the study; then, the medians and 95% CIs of the median responses within each group were calculated, and Mann–Whitney U tests were used to assess differences between groups. Pearson’s correlation coefficient (for normally distributed data) and Kendall’s rank correlation coefficient (for non-normally distributed data) were used as post hoc tests to clarify the relationships between baseline diet quality score, perceived healthiness, and liking of the meals. Hypothesis tests were not performed for the health-related outcomes, since these were not a primary objective of the study. Open-ended responses regarding dietary adherence and final evaluations of the study and diets as a whole were summarized and reported narratively in order to capture all responses. Missing data were handled using the listwise deletion method. All data analyses were performed using RStudio, R version 4.0.3 [47], the rstatix (v0.6.0; Kassambara, 2020), ggpubr (v0.4.0.999; Kassambara, 2020) and rcompanion packages (v2.3.26; Mangiafico, 2020).

## 3. Results

### 3.1. Participant Flow and Baseline Characteristics

**Figure 1:**
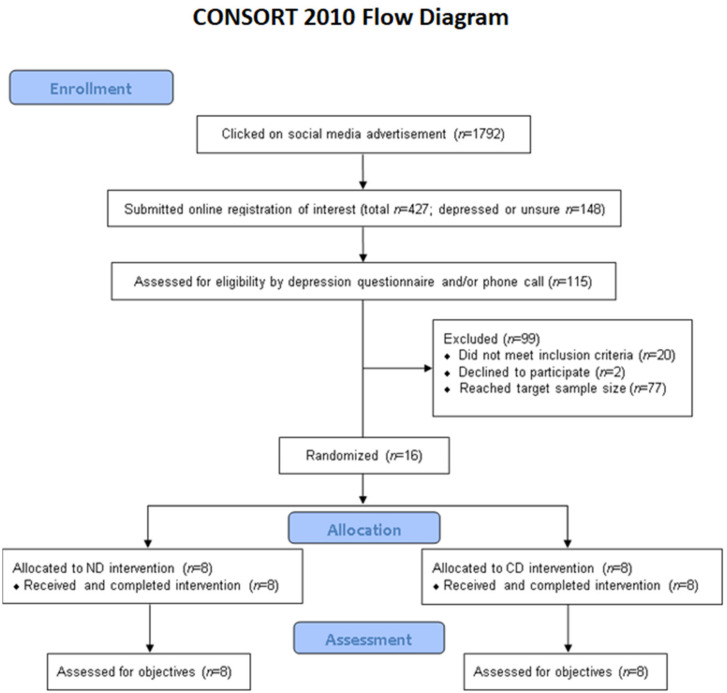
Consolidated Standards of Reporting Trials (CONSORT) flow diagram.

Figure 1 presents the CONSORT flow diagram of recruitment, enrollment, allocation, and assessment. Recruitment, enrollment, and the intervention took place in September 2020. Recruitment through social media was highly effective; the advertisement was active for 3 days and a total of 427 individuals completed an online registration of interest form. Of these, 115 individuals were further assessed for eligibility by a depression questionnaire and/or a phone call. Sixteen participants (10 depressed and 6 non-depressed) were enrolled and randomized in the study, as intended (ND, *n* = 8; CD, *n* = 8). All 16 participants completed the trial and were included in assessments. There was 100% compliance with the food pick-ups.

The baseline characteristics of all enrolled participants are presented in Table 2. The groups were well matched, indicating successful randomization. By design, the ND and CD groups had the same proportion of females to males and depressed to non-depressed participants. The median physical activity level was light to moderate. Among the depressed participants, the mean depression rating was in the range of moderate severity.

### 3.2. Adherence to the Diet Protocols

According to the daily participant questionnaires, in the ND group, six out of eight (75%) participants consumed no non-study foods during the intervention. This does not count the extra sandwich supplies that were provided and allowed. One participant ate two pieces of bread with margarine and salami on one occasion and one participant ate one cherry tomato and a half of a muffin. Seven out of eight participants in the ND group drank only permitted drinks and one participant drank half a serving of diet soda on one occasion.

In the CD group, four out of eight (50%) participants consumed no non-study foods. The non-study foods consumed by the remaining four participants consisted mostly of fruits and vegetables, which three participants reported that they missed. One of these ate approximately 500 g of extra fruits and vegetables on 6 out of 8 days. A handful of cashews, yogurt, and cottage cheese were the other non-study foods consumed in the CD group. Five out of eight participants in the CD group consumed only the permitted drinks. One participant drank one serving of diet soda, one drank two servings of juice, and one drank one serving of red wine (though alcohol consumption was permitted in amounts that they consumed habitually). Six out of eight participants in the CD group reported drinking all of the provided sugar-sweetened juice drink; one participant left two portions remaining and one participant left four portions remaining (out of eight portions total).

When asked “How easy was it for you to follow the diet protocol?” on the final questionnaire, on a scale of 1–7, the median response in the ND group was six: “It was easy” (95% CI: 3, 7), and the median response in the CD group was five: “It was pretty easy” (95% CI: 4, 6). When asked “How easy do you think it would be to participate in such a study, with meals recurring every 8 days for 8 weeks (but not have to fill out a questionnaire for the meals)?” the median response for the ND and CD groups was six: “It would be easy” (95% CI: 6, 7 and 5, 6, respectively).

### 3.3. Meal Evaluations

Table 3 presents the results of the meal evaluation questionnaires. Overall, the ratings for liking, sensory properties, and portion size were positive and quite similar between the diet groups. The meals in general, as well as the appearance and smell of the meals, were liked slightly more in the ND compared to the CD, though the differences were not statistically significant. In both groups, the smell matched the appearance of the meals, and the taste intensity and portion size were “just right”. The ND meals resulted in greater feelings of fullness and were perceived to be significantly healthier than the CD meals. Upon further analysis, a statistically significant negative correlation between baseline diet quality score and the perceived healthiness of the CD meals was identified (Pearson’s correlation coefficient r = −0.82, *p* = 0.012). Baseline diet quality score and liking of the CD meals were also negatively correlated, though not statistically significantly (r = −0.52, *p* = 0.19). Furthermore, there was a positive correlation between perceived healthiness of the meals and liking of the meals, which achieved statistical significance when both the CD and ND groups were included (Kendall’s rank correlation coefficient τ = 0.59, *p* = 0.004).

### 3.4. Health-Related Outcomes

The mean weight change was 0.1 (95% CI: −1.25, 1.45) kg in the ND group and −0.67 (95% CI: −2.03, 0.69) kg in the CD group (weight was estimated or measured by participants themselves and *n* = 7 per group due to missing data). Table 4 presents the self-rated health and activity assessments at baseline and follow-up. Although no statistical inference might be deduced, baseline estimates were similar between groups, with the exception that perceived general health status (EQ-5D VAS) was lower in the CD group compared to the ND group. Both groups experienced a reduction in depressive symptoms from baseline to follow-up; a greater mean reduction was observed in the ND compared to the CD group (five vs. three points, respectively, among depressed participants). Reductions in work and activity impairment and a slight improvement in general health according to EQ-5D index value were observed in both groups. In contrast, with regard to perceived general health status (EQ-5D VAS), there was a slight decrease in the ND group and an increase in the CD group. This may, in part, be explained by the GSRS results, which showed an increase in gastrointestinal symptom severity in the ND group and a decrease in the CD group, which was driven largely by indigestion symptoms (i.e., gas, bloating, etc.), as indicated by the indigestion sub-score. All health-related outcomes should be interpreted with caution since these were not the primary aim of the study and the confidence intervals were wide and overlapping, indicating a degree of uncertainty in the estimates.

### 3.5. Open-Ended Responses from the Final Questionnaire

Based on the written comments in the ND group, it was clear that all participants understood that they received a healthy diet. In response to the question “What was your overall impression of the diet?”, most of the comments in the ND group were positive. The aspects experienced as positive were the abundance of vegetarian meals; the newness, variety, freshness, and tastiness of the food; the convenience of not having to plan and shop for food; eating more regularly; and the feeling of contributing to important research. One person reported feeling more alert and happier and that they slept better. The negative aspects of the ND reported were the lack of variety of the breakfasts, the high fiber content, which caused gastrointestinal symptoms, the lack of flexibility and choice, and the fact that there was an excess of food at times. In terms of “what worked well”, participants reported that it was easy and convenient to transport, store, and heat up the food, with very little preparation required; it was easy to pick up and transport the food by bike; and they could choose when to eat their snacks. What worked less well was that some things were hard to measure, such as breakfast cereals, nuts, and berries, and therefore ran out; it was difficult to keep track of the various things that should be mixed together for the breakfast cereals; some packages leaked; fish bones were annoying; they got tired of the breakfasts and snacks that were repeated a few times; the abundance of beans and kale caused gut problems; and it required a lot of effort to adhere strictly to the protocol. Additional suggestions were to allow participants to have some choice in meals; and to have the same breakfast every day for simplicity. One participant reported that portions were too big, and they did not get hungry for the next meal, while another participant stated that they needed more food because, even though they got full after every meal, they got hungry in between. Regarding changes in how they felt, two participants reported having more energy, two reported feeling happier or better mentally, and one was both happier and had more energy. Four participants reported experiencing gut problems such as gas, pain, and diarrhea as a result of the diet; one reported feeling much better mentally after the second day, but due to the gut problems, which started on the third day, their mental health declined again; one reported gut problems that lasted for the entire intervention and another reported an improvement in symptoms over time. Participants’ final comments included feeling happy about losing weight and planning to continue eating as regularly as they did in the study.

Most of the participants in the CD group were aware that their diet was not very healthy, citing the lack of fruits and vegetables, and a high amount of sugar, refined grains, and meat. One participant, however, perceived the diet to be healthy, with the explanation that they “lost weight and became more alert”; this person also had the lowest possible baseline diet quality score. Besides the perception that the diet was not very healthy, the overall impression of the CD was that the food was good, tasty, and well-prepared from scratch. The weekend treats were appreciated, and participants felt well taken care of; however, there were difficulties with particular spices, breakfast was too sweet and it was sometimes hard to eat such a hearty breakfast first thing in the morning, and they were not used to eating so many times per day. Participants expressed their desire for more “color”, i.e., fruits and vegetables, some remarked that the salad went bad after a day or two. One participant stated that it was obvious that they were in the “control group”, which made it difficult to adhere to the diet. There were suggestions to avoid certain spices that not everyone likes, for example, coriander; to have more sauce; less cottage cheese; to be able to choose which meals to eat for lunch and dinner; to be informed whether participants were allowed to add salt or spices; to modify the instructions in order to accommodate participants with no microwave; and to have a “drive-through” for picking up food. With regard to changes in how they felt, half of the participants in the CD group reported feeling no different, but, among them, one participant commented that they would feel mentally and physically worse on a diet like this long-term (and that they missed chocolate). One participant reported being constipated. Two reported feeling mentally better due to feeling taken care of and not having to think about shopping and planning for food, though one of them felt nauseous one day and one often felt tempted to eat other things that they craved. One participant reported having more stamina and sleeping better; this was the same participant who perceived the diet to be healthy and reported having lost weight and being more alert.

Participants in both groups perceived that things worked well in the study in general, commenting that picking up the food went smoothly; instructions were clear; communication was efficient between the participants and the study staff when questions arose; and that the extra sandwich supplies provided were appreciated. Both groups commented that there were too many plastic containers and suggested the use of reusable containers; there was even a suggestion to have two cooler bags for each participant so that they could return one bag with the used containers and the new bag would be already filled up and ready to go when they arrived for pick-up. At least one participant in each group also commented that their food was picked up too late in the morning to eat their morning snack and that instructions should be provided in advance so that participants who have to go directly back to work or school would have everything they needed for the day’s meals on pick-up day, including supplies that may be at home. In general, participants in both groups expressed satisfaction and gratitude for having participated in the study.

## 4. Discussion

This was the first RCT to compare a healthy Nordic diet with a control diet in which all food was provided to participants of both groups for the entire intervention. Moreover, this was a pilot study of what may be the first proposed double-blind intervention investigating diet as a treatment for depression. Overall, the results were positive and promising and lay a good foundation for the future definitive RCT. The recruitment and retention rates were very good, and participant feedback was positive in general. Adherence to the diet protocol was very good in the ND group, and not as good in the CD group, mainly due to participants supplementing their diet with fruits and vegetables. Both groups reported that it was/would be fairly easy to adhere to/participate in the present study and the future 8-week study, although responses in the ND group were slightly more positive. Overall, the meals were liked and perceived to have good sensory properties in both diet groups, though to a somewhat greater extent in the ND group. The ND meals resulted in greater feelings of fullness, which is to be expected due to their substantially higher fiber content and somewhat larger portion size, owing to lower energy density, but both diets were perceived to have adequate portion sizes, on average. The logistics of the study went smoothly, according to both study staff and participants, and REDCap proved to be a useful and reliable tool for data collection, with the meal questionnaires providing the added bonus of reminding participants to eat regularly. Although this pilot study was not designed to detect statistically significant differences in health outcomes, five out of eight participants in the ND group reported feeling better physically and/or mentally as a result of the intervention, in contrast to three out of eight in the CD group (two of the latter reported that this improvement was due to feeling “taken care of”). Furthermore, the majority of health-related self-reported outcomes improved in both groups, with a greater improvement in depression in the ND group compared to the CD group.

The CD meals were perceived to be significantly and substantially less healthy than the ND meals, a finding that was also evident in the adherence data and the written comments. A degree of dissatisfaction was expressed by the CD participants in this regard, with one specifically commenting that it was obvious that they were in a control group and that it was therefore difficult to adhere to the diet. Moreover, post hoc analyses revealed a significant negative correlation between baseline diet quality and perception of the healthiness of the meals in the CD, as well as an overall significant positive correlation between perceived healthiness and liking of the CD and ND meals. If we assume that perception of healthiness is a marker of nutritional knowledge, the former correlation is in line with findings of a systematic review, which found a significant positive relationship between nutrition knowledge and diet quality, especially with regard to intake of fruits and vegetables [48]. Indeed, the participant who expressed the most dissatisfaction with the CD and adhered the least was one of the participants with the highest baseline diet score (6), and the participant who perceived the CD to be healthy and to feel better had the lowest baseline diet score (1). These findings indicate that, although participants were not informed of the presence of a control group and which diets were being investigated in the study, blinding was likely not as successful as intended, and that habitual diet quality plays an important role in this regard. Diet quality corresponding to the upper range of the accepted diet scores seems to have been underestimated, such that the CD was not an adequate match for these participants, as intended. Therefore, in the future definitive trial, the cutoff for the baseline diet score criteria should be lowered to include only participants with a fairly poor habitual diet. This would help ensure that the CD is a good match for participants’ habitual diet, which would therefore likely improve blinding, adherence, and treatment effects, since individuals with a poorer diet would be expected to benefit the most from a diet intervention. In addition to this modification, the quantity of vegetables and/or fruit in the CD should increase slightly, as even the participant with the lowest possible diet quality score who thought that the diet was healthy commented that they missed fruit and ate a few portions of fruit during the study. Indeed, the CD contained no fresh fruit, and the only sources of fruit were jam, yogurt, juice and apple sauce. Even with these proposed modifications, the differences between the two diets will remain substantial.

Although this study was not powered to detect differences in health outcomes, the fact that the majority of health-related self-report outcomes improved in the CD group suggests the presence of participant expectation bias, which implies a degree of success with participant blinding. With regard to work and activity impairment and perceived general health, the improvement was even greater in the CD group compared to the ND group. Thus, at least to a certain extent, double blinding may be achievable in diet interventions such as this, especially with the proposed modifications, as discussed.

Gastrointestinal symptoms, and indigestion in particular, increased in severity in the ND group; half of the participants reported an increase in symptoms of varying degrees. Some of these participants had mild gastrointestinal symptoms even at baseline, so exclusion criteria may need to be more carefully considered, even though the future definitive RCT is already planned to exclude individuals with gastrointestinal disorders and irritable bowel syndrome. However, it is common to experience gastrointestinal symptoms due to sudden and large increases in fiber intake, and these usually subside after approximately one week; one participant specifically stated that their symptoms decreased over time. Nevertheless, it is worth considering modifying the diet to improve digestibility, for example, by reducing specific gas-forming foods such as kale and beans, spreading out such foods across different days, sprouting or fermenting beans, and perhaps slightly reducing the total fiber content in the diet. These modifications may be even more necessary considering the proposed modification to reduce the baseline diet quality cutoff and may thereby help to improve the experience of the participants, increase study retention, and improve outcomes.

Some additional modifications for the future trial should be considered based on participant feedback, including using reusable food containers, providing greater quantities of items that have to be measured to avoid participants running out of them, allowing some choice of meals and/or flexibility with regard to which day and time the meals are eaten, and allowing snacks to be optional or saved for another time, since not everyone feels the need or desire to eat them. Providing different amounts of food depending on energy needs should also be considered in order to reduce food waste and ensure that those with higher energy needs receive enough food without having to eat an excessive number of extra sandwiches. The responses and written feedback regarding each meal will also be reviewed in detail to make decisions on specific meals that should be replaced or modified.

The limitations of the present pilot study include the short duration of 8 days, as the future RCT will be 8 weeks. However, the main reason for performing this pilot study was to ensure that all of the meals would be palatable and well-tolerated by participants in the future trial since the 8-day meal plan will be repeated seven times. Additionally, inclusion and exclusion criteria will be substantially more stringent in the future RCT for reasons of safety and to ensure the integrity of the findings; for example, clinically unstable medical disorders and various psychiatric disorders will be excluded and depression status and severity will be diagnosed and assessed by clinicians. Nevertheless, the MADRS-S is a well-validated and commonly used self-rated tool for assessing depression in clinical studies, and the baseline depression rating among depressed participants in the present study was in the range of moderate severity, which is reflective of the inclusion criteria in the future trial. Another potential limitation is that participants were recruited solely from social media. It is possible that this may have resulted in selection bias, although the response to the advertisement was substantial and represented a diversity of ages, depression severities, and ethnicities. Lastly, due to the pilot nature of this study, potential confounding factors such as alcohol consumption, medication use, physical activity and sleep were not considered in the assessment of depression or other health outcomes; these factors will be considered in the design and analyses of the future trial.

## 5. Conclusions

In summary, this pilot study demonstrates the feasibility and acceptability of an RCT comparing a healthy Nordic diet with a control diet in individuals with depression, in which all meals are provided to both groups. This study lays the foundation for a future 8-week RCT in which the aim will be to investigate whether a healthy Nordic diet improves depressive symptoms, as well as the biological mechanisms underlying this effect and potential factors that may predict treatment response. As this study also included non-depressed participants, the findings can be generalized, and a similar intervention can be applied in the treatment of other health conditions.

## Figures and Tables

**Table 1:** Diet compositions.

Food Group	Healthy Nordic Diet (ND)	Control Diet (CD)
Vegetables (excluding potatoes) and legumes	610 g/day	121 g/day
Fruit	303 g/day	29 g/day
Whole grain	115 g/day whole grains; no refined grains	27 g/day whole grains; mostly refined grains
Grains	55% barley, oat and rye	Mostly wheat
Added sugars	0 E%	11 E%
Dairy products	0.5% fat milk, 0.5% fat unsweetened yogurt, 17% fat cheese	1.5% fat milk, 2.5% fat sweetened yogurt, 28% fat cheese
Fats/oils	100% plant-based margarine, rapeseed oil, sunflower oil, olive oil (to a lesser extent)	Mostly butter; to a lesser extent rapeseed and other oils
Saturated fat	~8 E%	~16 E%
Vitamin D	>21 μg/day	>7 μg/day
Vitamin C	>191 mg/day	>60 mg/day
Iron	>16 mg/day	>~10 mg/day
Protein type for lunches and dinners		
Vegetarian	7 meals	3 meals
Poultry	5 meals	3 meals
Fish	4 meals (2 fatty)	1 meal (fatty)
Red meat (sausage not included)	0 meals	7 meals
Sausage	0 meals	2 meals

**Table 2:** Baseline characteristics of participants randomized to the healthy Nordic diet (ND) and control diet (CD).

		Total	ND (*n* = 8)	CD (*n* = 8)	*p*
Gender % female (by design)	% (*n*)	62.5% (10)	62.5% (5)	62.5% (5)	
Age	Median (IQR)	45 (22)	37 (17)	50 (15)	0.37
% Depressed (by design)	% (*n*)	62.5% (10)	62.5% (5)	62.5% (5)	
Frändin–Grimby Activity level (1-6, from least to most active)	Median (IQR)	3.5 (1)	3.5 (1)	3.5 (1)	0.865
Body weight (kg) (ND *n* = 7)	Mean (SD)	87.8 (17.3)	88.8 (16.1)	87 (19.4)	0.85
Diet quality score (1–12, from lowest to highest quality)	Mean (SD)	4.5 (1.4)	4.5 (1.2)	4.5 (1.7)	1
Total MADRS score (0–54, from least to most severe symptoms)	Mean (SD)	15.7 (9.9)	15 (10)	16.4 (10.5)	0.792
Total MADRS score, depressed only	Mean (SD)	22.2 (5.9)	21.8 (4.3)	22.6 (7.7)	0.845

IQR—interquartile range; SD—standard deviation; MADRS-S—Montgomery–Åsberg Depression Rating Scale, self-rated.

**Table 3:** Median (95% confidence interval (CI)) responses of meal evaluation questions in the healthy Nordic diet (ND) and control diet (CD) groups.

	ND (*n* = 8)	CD (*n* = 8)	*p*
How do you like the meal? (1 = Dislike very much, 7 = Like very much)	“Like” 6 (6, 7)	“Like somewhat/ Like” 5.5 (4, 6)	0.0713
What do you think of the meal’s appearance? (1 = Dislike very much, 7 = Like very much)	“Like” 6 (5, 7)	“Like somewhat/ Like” 5.5 (4, 6)	0.126
What do you think of the meal’s smell? (1 = Dislike very much, 7 = Like very much)	“Like” 6 (5, 7)	“Like somewhat” 5 (4, 5.5)	0.138
Does the smell of the food match the food you see on the plate? (1 = Doesn’t match at all, 5 = Matches very well)	“It matches” 4 (4, 5)	“It matches” 4 (4, 5)	0.747
What do you think of the taste intensity? (1 = Much too low, 5 = Much too high)	“Just right” 3 (3, 3)	“Just right” 3 (3, 3)	0.382
What did you think of the portion size? (1 = Much too small, 7 = Much too big)	“Just right” 4 (4, 4)	“Just right” 4 (4, 4)	0.382
How full do you feel after the meal? (0 = Not at all full, 100 = Have never felt so full)	73 (52, 81)	57 (35, 63)	0.0312
How healthy do you think the meal is? (0 = Not at all healthy, 100 = Very healthy)	83 (67, 95)	45 (28, 55)	0.0019

**Table 4:** Estimates of health-related assessments in the healthy Nordic diet (ND) and control diet (CD) groups at baseline and follow-up (8 days).

		ND (*n* = 8)		CD (*n* = 8)	
		Baseline	Follow-up	Baseline	Follow-up
MADRS-S total score (0–54, from least to most severe symptoms)	Mean (95% CI)	15 (7, 23)	11 (2, 20)	16 (8, 25)	13 (4, 22)
MADRS-S total score, depressed only	Mean (95% CI)	22 (16, 27)	17 (6, 28)	23 (13, 32)	20 (12, 27)
Frändin–Grimby Activity Scale (1–6, from least to most active)	Median (95% CI)	3.5 (3, 4)	4 (3, 4)	3.5 (3, 4)	3 (3, 4)
EQ-5D index value (0 = health as bad as dead, 1 = full health)	Mean (95% CI)	0.803 (0.686, 0.919)	0.838 (0.712, 0.964)	0.784 (0.677, 0.892)	0.801 (0.685, 0.917)
EQ-5D VAS (0 = Worst health imaginable, 100 = Best health imaginable)	Mean (95% CI)	73 (55, 90)	70 (53, 87)	59 (43, 75)	71 (61, 80)
WPAI work impairment (%)	Median (95% CI)	19 (0, 51)	15 (0, 20)	20 (0, 51)	10 (0, 35)
WPAI activity impairment (%)	Mean (95% CI)	30 (8, 52)	23 (2, 43)	28 (2, 53)	20 (3, 37)
GSRS total mean score (1 = no symptoms, 7 = very severe symptoms)	Mean (95% CI)	2.1 (1.4, 2.8)	2.5 (1.6, 3.4)	2.2 (1.3, 3.0)	1.9 (1.3, 2.5)
GSRS indigestion sub-score (1 = no symptoms, 7 = very severe symptoms)	Median (95% CI)	2.5 (1.5, 3.5)	3.5 (1.8, 4.3)	2.5 (1.5, 3.8)	1.6 (1, 2.5)

MADRS-S—Montgomery–Åsberg Depression Rating Scale, self-rated; EQ-5D-5L—EuroQol five-level generic health questionnaire; VAS—visual analog scale; WPAI—Work Productivity and Activity Impairment Questionnaire; GSRS—Gastrointestinal Symptoms Rating Scale. The mean was calculated for interval data that were normally distributed, without extreme outliers. The median was calculated for non-normally distributed or ordinal data. WPAI work impairment does not include the five participants who were unemployed (ND *n* = 6, CD *n* = 5).

## Data Availability

The data presented in this study are available upon reasonable request from the corresponding author. The data are not publicly available due to ethical and privacy restrictions, as well as General Data Protection Regulation (GDPR).
